# Analysis of Plasma Concentrations of Theophylline in Smoking and Nonsmoking Patients with Asthma

**DOI:** 10.3889/oamjms.2015.117

**Published:** 2015-11-14

**Authors:** Zlatica Goseva, Angelko Gjorcev, Biserka Jovkovska Kaeva, Elena Jovanovska Janeva, Irina Angelovska

**Affiliations:** *University Clinic of Pulmonology and Allergology, Faculty of Medicine, Ss Cyril and Methodius University of Skopje, Skopje, Republic of Macedonia*

**Keywords:** asthma, theophylline, plasma concentration, cigarette smoking, theophylline clearance

## Abstract

**BACKGROUND:**

While dosing theophylline in asthmatics, we should consider that a lot of medicines, substances, conditions and diseases affect the clearance of theophylline, such as smoking, macrolide antibiotics, barbiturates, oral contraceptives, heart and liver insufficiency, alcohol, calcium-antagonists, pneumonia, viral infections, hypoxemia, etc.

**AIM:**

The aim of the study is to investigate the concentrations of theophylline during the day in smoking and nonsmoking patients with asthma.

**MATERIAL AND METHODS:**

We have estimated the concentrations of theophylline 6 times daily by the HPLC method (Keith Muir, J Chromatography) in 20 smoking and 20 nonsmoking asthmatic patients, who were treated with theophylline sustained-release tablets 175 mg twice daily.

**RESULTS:**

In the first group of 20 nonsmoking patients we obtained constant therapeutic and optimal concentrations of theophylline. In the second group of 20 smoking asthmatics the concentration of theophylline in plasma, in 8pm and 8am the next day was very low.

**CONCLUSION:**

Because in smokers we have increased clearance and the decreased half- life of theophylline, and in order to prevent the night time life-threatening attacks, it is necessary to recommend maximal doses of theophylline, especially in the evening. According to the study, dosage should be individualized in order to optimize the treatment based on the measurement of theophylline concentration in plasma.

## Introduction

Asthma is a heterogeneous disease, usually characterized by chronic airway inflammation. It is defined by the history of respiratory symptoms such as wheeze, shortness of breath, chest tightness and cough that vary over time and in intensity, together with variable expiratory airflow limitation. Symptoms and airflow limitation may resolve spontaneously or in response to medication. Pharmacological therapy is an integral part and perhaps the most important part of all the measures included to control the asthma. The drugs that are used are divided into two groups. Anti-inflammatory or preventive medications “Controllers” are used to reduce the inflammation of the airways. Relieving or symptomatic medications “Relievers” are used to prevent the asthma attacks and the acute symptoms [1].

Methylxanthines (Theophylline) are Relievers medications, which are used in the treatment of asthma like bronchodilators [1]. Theophylline was first extracted from tea and synthesized chemically in 1895 and initially used as a diuretic. Its bronchodilator property was later identified, and it was introduced as a clinical treatment for asthma in 1922 [2].

Theophylline has become a third-line treatment as an add-on therapy in patients with poorly controlled asthma in step 2, 3 and 4, according to the actual version of the GINA guidelines (Global Initiative of Asthma), because inhaled beta 2 agonists are far more effective as bronchodilators, and inhaled corticosteroids have a greater anti-inflammatory effect [1]. Theophylline is used as an oral (rapid or slow-release tablets) for chronic treatment and intravenously for acute exacerbation of asthma [2, 3].

Theophylline is a weak nonselective inhibitor of phosphodiesterase (PDE) isoenzymes, which break down cyclic nucleotides in the cell, leading to increased intracellular concentrations of cAMP and cyclic guanosine monophosphate concentrations. Its main effect is to relax airway smooth muscle [2].

The Theophylline has demonstrated efficacy in attenuating the three cardinal features of asthma – reversible airflow obstruction, airway hyperresponsiveness, and airway inflammation [3]. The combination of inhaled corticosteroids and theophylline exerts a synergistic anti-inflammatory effect that improves asthma control and reduces asthma exacerbations [2, 4, 6].

There is a close relationship between the acute improvement in airway function and serum theophylline concentrations. Below 10 mg/L bronchodilator effects are small, and above 25 mg/L additional benefits are outweighed by side effects, so that the therapeutic range was usually taken as 10 to 20 mg/L, and is preferable to redefine the therapeutic range as 5 to 15 mg/L, which will avoid the risk of side-effects like anorexia, nausea, headache and sleep disturbance. Altered mood and behavior are sufficiently common to limit theophylline use in young children. Theophylline may also aggravate underlying gastro-oesophageal reflux. The dose of theophylline required to achieve therapeutic concentrations varies among patients, largely because of differences in clearance. Theophylline is rapidly and completely absorbed, but there are large interindividual variations in clearance, due to differences in its hepatic metabolism. Theophylline is metabolized in the liver by the cytochrome P450 microsomal enzyme system, and a large number of factors may influence hepatic metabolism. Theophylline is predominantly metabolized by CYP1A2. Increased clearance is seen in children (1–16 yr) and in cigarette and marijuana smokers. Concurrent administration of phenytoin, phenobarbitone, or rifampicin, which increases P450 activity, increases metabolic breakdown, so that higher doses may be required. Reduced clearance is found in liver disease, pneumonia, and heart failure, and doses need to be reduced to one-half and plasma levels monitored carefully [2]. Decreased clearance is also seen with several drugs, including erythromycin, quinolone antibiotics (ciprofloxacin, but not ofloxacin), allopurinol, cimetidine (but not ranitidine), serotonin uptake inhibitors (fluvoxamine), and the 5-lipoxygenase inhibitor zileuton, all of which interfere with CYP1A2 function. Thus, if a patient on maintenance theophylline requires a course of erythromycin, the dose of theophylline should be halved. Although there is a similar interaction with clarithromycin, there is no interaction with azithromycin. Viral infections and vaccinations (influenza immunizations) may also reduce clearance, and this may be particularly important in children. Because of these variations in clearance, individualization of theophylline dosage is required, and plasma concentrations should be measured 4 h after the last dose with slow release preparations, when steady state has usually been achieved [2].

Cigarette smoking remains highly prevalent in some countries. It can affect drug therapy by both pharmacokinetic and pharmacodynamic mechanisms. The mechanism involved in most interactions between cigarette smoking and drugs involves the induction of metabolism. Drugs for which induced metabolism because of cigarette smoking may have clinical consequence include theophylline [7].

In the determination of the dosing strategy of theophylline in asthmatic patients, it is important to consider the fact that a big number of medications, substances, conditions and diseases influence the clearance of the drug. This fact has serious therapeutic implications. For example, the smoking cause increase of theophylline clearance by 58–100% and decrease its half life (T/2) by 63% in the smokers organism compared with nonsmokers. This is because it is highly metabolized by CYP1A2. Smoking cessation for 1 week reduces the elimination of theophylline by 35% [8]. This implies a necessity of careful planning of the dosage and of frequent sampling of theophylline in plasma.

The aim of our study is to investigate the concentration of theophylline during the day using the comparative analysis in smoking and nonsmoking patients with asthma. The aim of study is to show that dosage of theophylline should be individualized in order to optimize the treatment of patient with asthma.

## Material and Methods

In our investigation were included 40 patients with diagnosed asthma. They were divided in two groups as follows: 1) First group were 20 nonsmoking asthmatics; 2) Second group were 20 patients with asthma who were smokers. All patients were treated with combined therapy with inhaled corticosteroids and long acting beta 2 agonist (ICS/LABA) and theophylline sustained-release tablets 175 mg twice daily. We have investigated the plasma level of theophylline by HPLC method, Keith Muir, J Chromatography [9]. The samples of blood were taken in 8 am and 10 am; in 2 pm, 8 pm, and 12 pm and then in 8 am the next morning. We used samples of 5 ml heparinized blood from the cubital vein, from which we extracted 2 ml of plasma by centrifugation.

## Results

**Figure 1:**
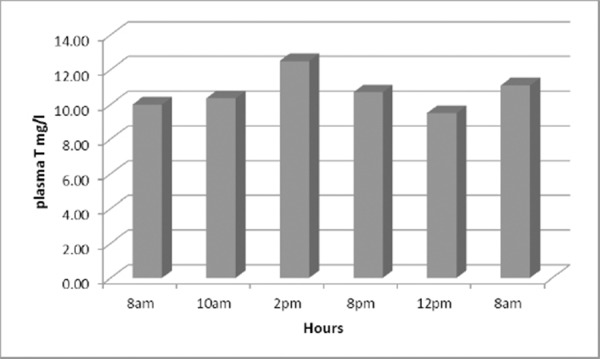
*Levels of theophylline obtained five times daily in nonsmoking asthmatic*.

Our results have shown that in the first group of nonsmoking asthmatics we achieved therapeutic optimal and constant concentrations of the medication theophylline (Fig. 1).

**Figure 2:**
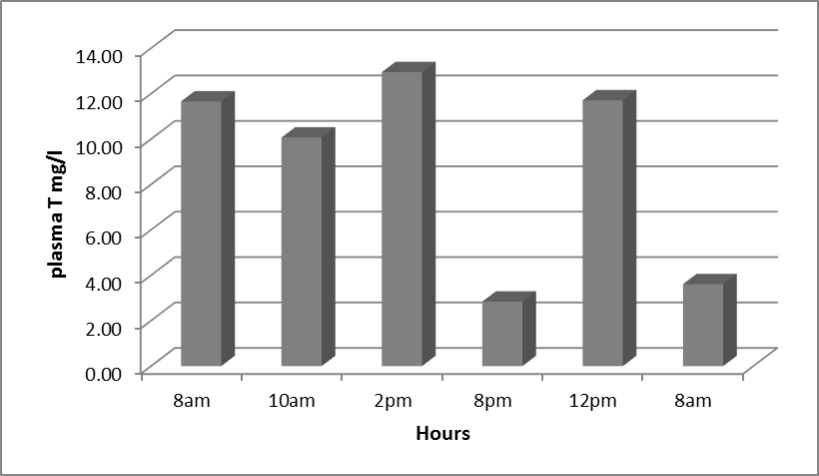
*Levels of theophylline obtained five times daily in smoking asthmatics*.

In our investigation we have found that in the second group of asthmatics who were smokers the theophyllinemia in 8 pm and 8 am on the second morning was extremely low (Fig. 2).

In the group of 20 smoking asthmatics the concentration of theophylline in blood, in 8 pm and 8am the next day was very low. These results suggest the need to provide and to prescribe theophylline therapy early in the morning and early in the evening.

## Discussion

The impact of cigarette smoking needs to be considered in planning and assessing responses to drug therapy [7]. Also because of its narrow therapeutic range, dosage must be individualized in order to optimize the treatment based on the measurement of theophylline concentration in serum [3]. In our study nonsmoking asthmatics have therapeutic optimal and constant concentrations of the medication theophylline, but in the second group of asthmatics who were smokers, the theophyllinemia in 8 pm and 8 am on the second morning of our study, was extremely low. In literature it is known that smoking increase theophylline metabolism [8, 10]. Thomson N.C and al. in their study explain that cigarette smoking increase the clearance of drugs by induction of several metabolizing enzymes. Cytochrome P450 1A2 is responsible for the metabolism of theophylline and clearance is increased by 60-100%in smokers compared with nonsmokers [8]. According to this explanation in our study we also noticed that the smoking cause increase of theophylline clearance. Because of this finding we suggest more frequently control of blood concentrations of theophylline. Barnes J.P. in his study about theophylline says that efficacy is related to blood concentration, which are determined mainly by hepatic metabolism, which may be increased or decreased [2]. Low dose of theophylline may achieve control of asthma comparable to a low dose of inhaled corticosteroids [6]. In asthmatics patients low dose theophylline reduce eosinophils and other inflammatory markers, inhibits the eosinophilia induced by an inhaled allergen and reduce the expression of cytokines such as interleukin-5 [6]. But, in smoking asthmatics in addition to eosinophilic airway inflammation, smoking induced neutrophilic airway inflammation [11, 12]. Therefore, smokers taking a medication that interacts with smoking may require higher dosages then nonsmokers [13]. In literature it is known that smokers with asthma have a reduced response to inhaled corticosteroid therapy [10, 14-17]. Alternative or additional therapies to inhaled corticosteroids are needed for individuals with asthma who are unable to quit smoking [14]. So, theophylline could be providing as additional medication to inhaled corticosteroids in smoking asthmatics.

In conclusion, in our study in nonsmoking asthmatics we achieved therapeutic optimal and constant theophylline concentrations, which lead to subsequent therapeutic effects. In the smoking asthmatics in 8 pm and 8 am on the second morning of the examination, the concentrations of theophylline were extremely low. We suggest that this low concentration is result of the increased clearance and the decreased T/2 of theophylline in smokers. Because of the increased clearance and the decreased half-life of theophylline in smokers, and in order to prevent the night time life-threatening attacks, it is necessary to recommend maximal doses of theophylline, especially in the evening. Having in mind these facts, it is important for smoking asthmatics, to provide and to prescribe theophylline therapy early in the morning and early in the evening and to control the plasma concentrations of theophylline more frequently.
